# Is albumin administration in the acutely ill associated with increased mortality? Results of the SOAP study

**DOI:** 10.1186/cc3895

**Published:** 2005-11-07

**Authors:** Jean-Louis Vincent, Yasser Sakr, Konrad Reinhart, Charles L Sprung, Herwig Gerlach, V Marco Ranieri

**Affiliations:** 1 Department of Intensive Care, Erasme Hospital, Free University of Brussels, Route de Lennik 808, 1070 Brussels, Belgium; 2 Department of Anaesthesiology and Intensive Care, Friedrich-Schiller-University, Erlanger Allee 101, 07747, Jena, Germany; 3 Department of Anaesthesiology and Critical Care Medicine, Hadassah Hebrew University Medical Center, P.O.B. 12000, 91120 Jerusalem, Israel; 4 Department of Anaesthesiology and Intensive Care, Vivantes-Klinikum Neukölln, Rudower strasse 48, 12313 Berlin, Germany; 5 Department of Anaesthesiology and Intensive Care, S Giovanni Battista Hospital, University of Turin, Corso Dogliotti 14, 10126 Torino, Italy

## Abstract

**Introduction:**

Albumin administration in the critically ill has been the subject of some controversy. We investigated the use of albumin solutions in European intensive care units (ICUs) and its relationship to outcome.

**Methods:**

In a cohort, multicenter, observational study, all patients admitted to one of the participating ICUs between 1 May and 15 May 2002 were followed up until death, hospital discharge, or for 60 days. Patients were classified according to whether or not they received albumin at any time during their ICU stay.

**Results:**

Of 3,147 admitted patients, 354 (11.2%) received albumin and 2,793 (88.8%) did not. Patients who received albumin were more likely to have cancer or liver cirrhosis, to be surgical admissions, and to have sepsis. They had a longer length of ICU stay and a higher mortality rate, but were also more severely ill, as manifested by higher simplified acute physiology score (SAPS) II and sequential organ failure assessment (SOFA) scores than the other patients. A Cox proportional hazard model indicated that albumin administration was significantly associated with decreased 30-day survival. Moreover, in 339 pairs matched according to a propensity score, ICU and hospital mortality rates were higher in the patients who had received albumin than in those who had not (34.8 versus 20.9% and 41.3 versus 27.7%, respectively, both *p* < 0.001).

**Conclusion:**

Albumin administration was associated with decreased survival in this population of acutely ill patients. Further prospective randomized controlled trials are needed to examine the effects of albumin administration in sub-groups of acutely ill patients.

## Introduction

Albumin administration in the critically ill is controversial and hotly debated, despite having been accepted and widely used for more than 50 years. A meta-analysis by the Cochrane group [1] published 5 years ago first put light to this fire, showing an increased mortality in patients treated with albumin in their analysis of 30 randomized controlled trials including 1,419 randomized patients. An accompanying editorial even suggested that, based on these results, "the administration of albumin should be halted" [2]. The Cochrane analysis was criticized by a later meta-analysis [3] because it excluded, for various reasons, several trials that had shown reduced mortality rates with albumin administration. When more studies were included into the meta-analysis, an adverse effect of albumin on mortality could no longer be demonstrated [3]. Both analyses, however, have the limitation that the inclusion criteria were very broad and the fluid regimen very different among the included trials. In a recent randomized controlled study (the Saline versus Albumin fluid Evaluation (SAFE) study) providing data on nearly 7,000 patients randomized to receive either albumin or normal saline as resuscitation fluid, there was no difference in outcome between the two groups [4].

While randomized controlled trials such as the SAFE study provide strong evidence for or against an intervention, epidemiological studies allowing for multivariable analyses can provide useful additional information on the current use of albumin and on associated outcomes. The Sepsis Occurrence in Acutely ill Patients (SOAP) study did exactly this to determine current intensive care unit (ICU) practice and the effects of that practice on outcomes for various topics, including administration of albumin.

## Methods

### Study design

The SOAP study was a prospective, multicenter, observational study designed to evaluate the epidemiology of sepsis as well as other characteristics of ICU patients in European countries and was initiated by a working group of the European Society of Intensive Care Medicine. Institutional recruitment for participation was by open invitation from the study steering committee. As this epidemiological observational study did not require any deviation from routine medical practice, institutional review board approval was either waived or expedited in participating institutions and informed consent was not required. All patients older than 15 years admitted to the participating centers (see Acknowledgements below for a list of participating countries and centers) between 1 May and 15 May 2002 were included. Patients were followed up until death, hospital discharge, or for 60 days. Those who stayed in the ICU for less than 24 hours for routine postoperative observation were excluded.

### Data management

Data were collected prospectively using preprinted case report forms. Detailed explanations of the aim of the study, instructions for data collection, and definitions for various important items were available for all participants via the Internet [5] before starting data collection and throughout the study period. The steering committee processed all queries during data collection.

Data were entered centrally by medical personnel using the SPSS v11.0 for Windows (SPSS Inc, Chicago, IL, USA). A sample of 5% of data was re-entered by a different encoder and revised by a third; a consistency of more than 99.5% per variable and 98.5% per patient were observed during the whole process of data entry. In cases of inconsistency, data were verified and corrected. Daily frequency tables were revised for all variables and the investigators were queried when data values were either questionable or missing for required fields. There was no data quality control at the data collection level.

Data collection on admission included demographic data and comorbidities. Clinical and laboratory data for the simplified acute physiology (SAPS) II score [6] were reported as the worst value within 24 hours after admission. Microbiological and clinical infections were reported daily as well as the antibiotics administered. A daily evaluation of organ function, based on a set of laboratory and clinical parameters according to the sequential organ failure assessment (SOFA) score [7], was performed, with the most abnormal value for each of the six organ systems (respiratory, renal, cardiovascular, hepatic, coagulation, and neurological) being collected on admission and every 24 hours thereafter. For a single missing value, a replacement was calculated using the mean value of the results on either side of the absent result. When the first or last values were missing the nearest value was carried backward or forward, respectively. When more than one consecutive result was missing, it was considered to be a missing value in the analysis. Overall, missing data represented less than 6% of collected data, and 2% of these values were replaced.

### Definitions

Infection was defined as the presence of a pathogenic microorganism in a sterile milieu (such as blood, abscess fluid, cerebrospinal or ascitic fluid), and/or clinically documented infection, plus the administration of antibiotics. Sepsis was defined according to the American College of Chest Physicians/Society of Critical Care Medicine (ACCP/SCCM) consensus conference definitions, by infection plus two systemic inflammatory response syndrome (SIRS) criteria [8]. Organ failure was defined as a SOFA score >2 for the organ in question [9]. Severe sepsis was defined by sepsis plus at least one organ failure. Mean fluid balance was calculated as the total fluid balance during the ICU stay divided by the duration of ICU stay in days.

### Statistical methods

Data were analyzed using SPSS v11.0 for Windows (SPSS Inc, Chicago, IL, USA). Descriptive statistics were computed for all study variables. The Kolmogorov-Smirnov test was used and stratified distribution plots were examined to verify the normality of distribution of continuous variables. Nonparametric tests of comparison were used for variables evaluated as not normally distributed. Difference testing between groups was performed using the two-tailed *t* test, Mann-Whitney *U* test, Chi square test, and Fisher exact test as appropriate. To determine the relative hazard of death due to albumin administration, a Cox proportional hazard model [10] was constructed with time to death, right censored at 30 days as the dependent factor and, as independent factors, age, sex, trauma, comorbidities on admission, SAPS II score on admission, the timing of onset of albumin administration, use of other colloids and blood products (red blood cells, fresh frozen plasma), and the mean fluid balance, the degree of organ failure assessed by the SOFA score, procedures (mechanical ventilation, pulmonary artery catheter, renal replacement therapy), and the presence of sepsis syndromes on admission in patients who did not receive albumin and at onset of albumin administration in those who did, were also included as independent variables. Covariates were selected and entered in the model if they attained a *p* value <0.2 on a univariate basis. Seven countries were included in the model, six being identified as a risk of decreased survival and one with a favorable prognosis compared with the others. A forward stepwise approach was performed. Only significant variables were retained in the final model. The time dependent covariate method [10] was used to check the proportional hazard assumption of the model; an extended Cox model was constructed, adding interaction terms that involve time (for example, time dependent variables) computed as the byproduct of time and individual covariates in the model (time*covariate); individual time dependent covariates were introduced one by one and in combinations in the extended model, none of which was found to be significant (Wald chi-square statistic). The Cox proportional hazard model was reconstructed, stratifying patients according to the presence or absence of trauma or severe sepsis.

Propensity scores [11] were obtained through forward stepwise logistic regression of patients' characteristics on albumin infusion status [11-14], that is, albumin administration as the dependent factor (Table 1). Variables were entered into the model and removed at a cutoff *p* value of 0.2. The propensity score was calculated as the probability based upon the final model. A greedy matching technique [15] was used to match individual patients who received albumin at any time with individual patients without albumin based on propensity scores. The best-matched propensity score was identical to five digits. Once a match was made, the control patient was removed from the pool. This process was then repeated using four-digit matching, then three-digit matching, and so on. The process proceeded sequentially to a single-digit match on propensity score. If a match was not obtained at this point, the patient who had received albumin was excluded. Baseline characteristics were compared between the two matched groups without comparing mortality and the process was repeated by adding interactions to the logistic regression model involving the unmatched covariates, including replacing it by its square or multiplying two unmatched covariates [12]. Kaplan Meier survival curves were plotted and compared using the signed Log Rank test in the propensity score matched pairs. Another Cox regression model was constructed as described above in the group of matched pairs involving the propensity score as a covariate. All statistics were two-tailed and a *p* value <0.05 was considered to be statistically significant.

## Results

**Figure 1:**
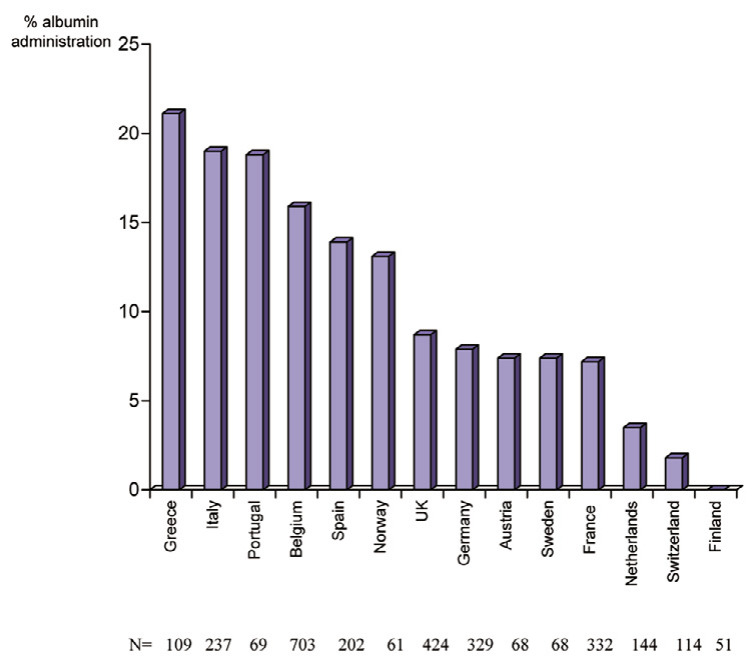
Bar chart representing the percentage of patients receiving albumin infusions in the various contributing countries. Only countries that included more than 50 patients are considered.

Of 3,147 patients, 354 (11.2%) received albumin and 2,793 (88.8%) did not. Figure 1 represents the proportion of patients who received albumin in the 14 most represented countries. In general, albumin administration was more commonly used in the south of Europe. Albumin was administered during the first 24 hours following admission in 157 (44.4%) of those who received it; only 34 patients (7.6%) received albumin after 7 days of admission.

Clinical data are presented in Table 2. Patients who received albumin had the same mean age, but were more likely to have cancer or liver cirrhosis, to be a surgical admission, and to have sepsis than the patients who did not receive albumin. They had a longer length of ICU stay and a higher ICU mortality rate (35 versus 16%, *p* < 0.001), but were also more severely ill, as manifested by higher SAPS II and SOFA scores than the other patients. At the onset of albumin administration (Table 3), these patients had a higher degree of organ dysfunction failure as manifested by higher SOFA scores and higher incidence of sepsis and invasive procedures (mechanical ventilation, pulmonary artery catheterization, and renal replacement therapy) compared with these factors on admission in patients who never received albumin during the ICU stay.

**Figure 2:**
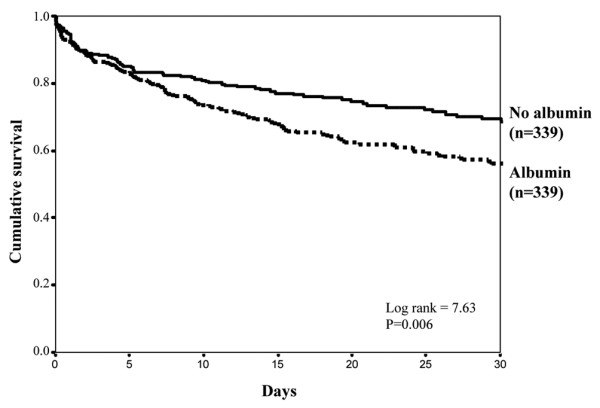
Kaplan-Meier survival curves in patients who received albumin (lower curve) and their propensity matched pairs without albumin administration.

In the Cox proportional hazard model, albumin administration was independently associated with a lower 30-day survival (relative hazard 1.57, 95% confidence interval (CI) 1.11–2.22, *p* = 0.012; Table 4). Albumin remained an independent risk of lower 30-day survival when stratifying for trauma (n = 254) or severe sepsis (n = 765) (Table 5). Moreover, in 339 pairs matched according to a propensity score, ICU (34.8 versus 20.9%, *p* < 0.001) and hospital (41.3 versus 27.7%, *p* < 0.001) mortality rates were higher in patients who received albumin than in those who did not; the survival curves are shown in Figure 2. In these matched pairs, albumin administration was associated with a decreased 30-day survival in a multivariable Cox proportional hazard analysis (relative hazard 1.57, 95% CI 1.19–2.07, *p* = 0.001; Table 4). Table 6 shows the baseline characteristics of the propensity-matched patients on admission on the basis of age, gender, comorbidities, type of admission, SAPS II and SOFA scores, procedures, and sepsis syndromes. Propensity scores were also associated with a decreased 30-day survival, both in the whole population and in the matched pairs.

## Discussion

In this observational study, patients who received albumin had higher ICU and hospital mortality rates than those who did not. This may be expected, as albumin administration is generally added to resuscitative fluids in very ill patients or patients with hypoalbuminemia and/or edema. Hypoalbuminemia itself is an independent predictor of an adverse outcome [16-18]. In the present study, patients who received albumin were also more severely ill, with a higher frequency of cancer, liver cirrhosis, and sepsis, and significantly higher SAPS II scores. Independent of albumin administration, these patients, therefore, had a higher risk of death than patients who did not receive albumin. We applied two different methods to control for these confounding factors. Firstly, we included the confounding variables in a Cox proportional hazard model. Secondly, we produced unbiased estimators of the effects of albumin administration on mortality rates by using a propensity analysis. In both analyses, the mortality rates after adjustment for confounding factors were still higher in patients who received albumin than in those who did not. Therefore, the administration of albumin was associated with higher mortality independent of those comorbid conditions included in our statistical models.

Although a prospective, controlled randomized clinical trial is, of course, the optimal means of demonstrating cause and effect, epidemiological studies with adequate multivariable analysis can provide valuable information. A similar approach has been taken to show that aspirin administration may reduce complications after coronary artery bypass grafting [19]. One must, however, remember that a multivariable analysis cannot take all factors into account, so that other unidentified factors in the patients who received albumin may have influenced the results. Nevertheless, many factors, including comorbid diseases, were included in the analysis due to the epidemiological nature of this study. Regional factors may also influence results and, indeed, there were considerable regional variations, with albumin generally being used more commonly in the south of Europe; however, we corrected for regional differences in our multivariable model.

The SOAP study was not originally designed to specifically address questions regarding albumin administration in the ICU. This analysis, therefore, has some limitations in addition to those of a multivariable analysis. First, the indications for albumin administration were not recorded. Second, serum albumin levels were not measured and it thus remains unclear whether albumin levels were successfully corrected in patients treated with albumin. Indeed, there are data suggesting that the use of albumin in patients with hypoalbuminemia may be beneficial. In a recently published meta-analysis [16], nine studies addressing morbidity in critically ill patients after correction of hypoalbuminemia were identified. There was a trend towards reduced morbidity in patients where hypoalbuminemia was corrected (odds ratio 0.74; 95% CI 0.41–1.60). The meta-analysis also suggested that albumin levels need to reach more than 30 g/l before albumin replacement becomes effective [16]; only four of the nine studies achieved this goal. It should be pointed out that three of the four studies were undertaken in pediatric patients. Another recent meta-analysis noted a trend towards reduced morbidity in hypoalbuminemic patients who received albumin (relative risk 0.92; 95% CI 0.77–1.08) [20]. Nevertheless, it remains unproven whether an improvement in morbidity translates into an improvement in survival.

The reason for an increased mortality in patients who received albumin cannot be identified from our study. Albumin has well-recognized, potentially important functions in the critically ill, including maintenance of colloid oncotic pressure, binding capacity for drugs and other substances, and scavenging of oxygen free radicals [21]. Starling's principle may not appropriately reflect the microcirculation in critically ill patients, however, especially under conditions of capillary leakage, as may happen in sepsis or burns [22]. Other possible negative effects of albumin administration may include myocardial depression due to decreased ionic calcium [23], and impaired renal function [24,25]. Furthermore, albumin has anti-thrombotic properties that might be detrimental in some patients [1,26].

The recently completed, randomized controlled SAFE study [4] showed no differences in outcome in critically ill patients requiring fluid repletion who were treated with 4% albumin compared to those treated with saline. The SAFE study was without doubt a well-conducted study that answered adequately the question it asked, that is, that in a heterogeneous population of critically ill patients albumin does not seem to have harmful effects. However, albumin was given, often transiently, as part of a fluid challenge and a 4% albumin solution was used. Therefore, a number of patients received only small amounts of albumin that were unlikely to influence outcome.

## Conclusion

Albumin may indeed be safe when used as a resuscitation fluid (as shown by the SAFE study), but our results suggest that it may not be safe all of the time in all critically ill patients. We believe further studies, such as the present, are needed to generate hypotheses and encourage further research to fully clarify the role of albumin in our ICUs.

## Key messages

• 	In this observational study of 3,147 patients, albumin administration was independently associated with a lower 30-day survival, using a Cox proportional hazard model.

• 	Moreover, in 339 pairs matched according to a propensity score, ICU and hospital mortality rates were higher in patients who received albumin than in those who did not.

• 	While albumin administration may be safe in patients requiring fluid for intravascular volume depletion, these results suggest it may not be harmless in all ICU patients.

## Abbreviations

CI = confidence interval; ICU = intensive care unit; SAFE = saline versus albumin fluid evaluation; SAPS = simplified acute physiology score; SOAP = sepsis occurrence in acutely ill patients; SOFA = sequential organ failure assessment.

## Competing interests

JLV has received research grants from PPTA. The other authors declare that they have no competing interests.

## Authors' contributions

JLV and YS participated in the design of the study. All authors contributed to data collection. YS performed the statistical analyses. JLV and YS drafted the manuscript. KR, CLS, HG, VMR revised the article. All authors read and approved the final manuscript.

## Figures and Tables

**Table 1:** Propensity score model

	Coefficient	SEM	Wald	Odds ratio (95% CI)	*p *value
SOFA score^a^	0.078	0.016	22.78	1.08 (1.05–1.12)	<0.001
HES administration^b^	0.591	0.129	21.10	1.81 (1.40–2.32)	<0.001
RBC transfusion^b^	1.296	0.134	93.03	3.65 (2.81–4.76)	<0.001
Cirrhosis	0.796	0.239	11.10	2.22 (1.39–3.54)	0.001
Medical admission	-0.407	0.132	9.47	0.67 (0.51–0.86)	0.002
Cancer	0.451	0.167	7.32	1.57 (1.13–2.18)	0.007
Sepsis^a^	0.332	0.133	6.24	1.39 (1.074–1.81)	0.012
Hemofiltration^a^	0.380	0.292	1.69	1.46 (0.83–2.59)	0.193
Hemodialysis^a^	0.525	0.368	2.04	1.69 (0.82–3.48)	0.154
Constant	-0.591	0.543	1.19	NA	0.276

The basic model used to determine the propensity score was a multivariable, forward stepwise, logistic regression analysis with albumin administration as the dependent factor. ^a^On the day of onset of albumin administration in the albumin group and on admission in other patients. ^b^At any time during intensive care unit stay. CI, confidence interval; HES, hydroxyethyl starch; RBC, red blood cell; SEM, standard error of mean; SOFA, sequential organ failure assessment.

**Table 2:** Characteristics of the study group

	All patients (n = 3,147)	Stratifying according to albumin administration		
		Albumin (n = 354)	No albumin (n = 2,793)	*p *value

Age, mean ± SD^a^	61 ± 17	62 ± 15	60 ± 18	0.15
Male (%)^b^	1,920 (61.7%)	219 (62.4)	1,701 (61.6)	0.776
Chronic diseases (%)				
COPD	340 (10.8)	38 (10.7)	302 (10.8)	0.964
Cancer	415 (13.2)	66 (18.7)	349 (12.5)	<0.001
Heart failure	307 (9.8)	42 (11.9)	265 (9.5)	0.156
Diabetes	226 (7.2)	29 (8.2)	197 (7.1)	0.434
Liver cirrhosis	121 (3.8)	32 (9.0)	89 (3.2)	<0.001
Hematologic cancer	69 (2.2)	13 (3.7)	56 (2.0)	0.053
HIV/AIDS	26 (0.9)	6 (1.7)	20 (0.7)	0.18
Surgical admission (%)	1,388 (44.1)	218 (61.6)	1,170 (41.9)	<0.001
SAPS II score, mean ± SD	36.5 ± 17.1	41.5 ± 17.3	35.9 ± 17.0	<0.001
Admission SOFA score, mean ± SD	5.1 ± 3.8	6.9 ± 3.9	4.9 ± 3.8	<0.001
Infection (%)	1,177 (37.4)	225 (63.6)	952 (34.1)	<0.001
On admission	777 (24.7)	140 (39.5)	637 (22.8)	<0.001
Severe sepsis (%)	930 (29.6)	202 (57.1)	728 (26.1)	<0.001
On admission	552 (17.5)	112 (31.6)	440 (15.8)	<0.001
Septic shock (%)	462 (16.5)	144 (40.7)	318 (11.4)	<0.001
On admission	243 (7.7)	62 (17.5)	181 (6.5)	<0.001
ICU stay, median (IQ)	3.0 (1.7–6.9)	8.0 (3.1–17.8)	2.9 (1.6–6)	<0.001
Hospital stay, median (IQ)^c^	15 (7–32)	27 (12–49)	14 (7–29)	<0.001
ICU mortality (%)^d^	583 (18.5)	125 (35.3)	458 (16.4)	<0.001
Hospital mortality (%)^c^	747 (23.7)	147 (41.5)	600 (21.3)	<0.001

^a^Nine missing. ^b^Thirty-five missing. ^c^Thirty-nine missing. ^d^One missing. ICU, intensive care unit; IQ, interquartile range; SAPS, simplified acute physiology score; SD, standard deviation; SOFA, sequential organ failure assessment.

**Table 3:** Comparison of patients who received albumin and those who did not

	All patients (n = 3,147)	Albumin (n = 354)	No albumin (n = 2,793)	*p *value
SOFA score, mean ± SD	5.2 ± 3.9	7.2 ± 4.2	4.9 ± 3.8	<0.001
Sepsis syndromes (%)				
Sepsis	765 (24.3)	128 (36.2)	637 (22.8)	<0.001
Severe sepsis	549 (17.4)	109 (30.8)	440 (15.8)	<0.001
Septic shock	241 (7.7)	60 (16.9)	181 (6.5)	<0.001
Procedures (%)				
Mechanical ventilation	1,853 (58.9)	269 (76.0)	1,584 (56.7)	<0.001
Pulmonary artery catheter	378 (12.0)	74 (20.9)	304 (10.9)	<0.001
Hemofiltration	82 (2.6)	23 (6.5)	59 (2.1)	<0.001
Hemodialysis	57 (1.8)	13 (3.7)	44 (1.6)	0.005
Organ failure (%)				
Respiratory	705 (22.4)	111 (31.4)	594 (21.3)	<0.001
Cardiovascular	784 (24.9)	156 (44.1)	628 (22.5)	<0.001
Coagulation	158 (5.0)	42 (11.9)	116 (4.2)	<0.001
Hepatic	89 (2.8)	19 (5.4)	70 (2.5)	0.002
Renal	562 (17.9)	76 (21.5)	486 (17.4)	0.060
Neurological	681 (21.6)	71 (20.1)	610 (21.8)	0.443

Sequential organ failure assessment (SOFA) score, sepsis syndromes, procedures, and organ failure (SOFA > 2) in patients who did and did not receive albumin compared with admission values for patients who did not receive albumin.

**Table 4:** Cox proportional hazard model with time to death, right censored at 30 days, as dependent factor

	All patients (n = 3,147)		Propensity matched patients (n = 678)	
	Relative hazard (95% CI)	*p *value	Relative hazard (95% CI)	*p *value

SAPS II score^a^	1.04 (1.04–1.05)	<0.001	1.02 (1.01–1.03)	<0.003
SOFA score^b^	1.06 (1.03–1.08)	<0.001	1.05 (1.01–1.10)	0.032
Medical admission	1.78 (1.25–2.21)	<0.001	2.33 (1.63–3.31)	<0.001
Age	1.01 (1.01–1.02)	<0.001	1.02 (1.01–1.03)	0.003
Cirrhosis	2.23 (1.68–2.95)	<0.001	1.91 (1.23–2.98)	0.004
Mean fluid balance	1.30 (1.24–1.37)	0.001	1.30 (1.19–1.42)	<0.001
Hemofiltration	1.25 (1.04–1.50)	0.019	-	-
Albumin administration^b^	1.57 (1.11–2.22)	0.012	1.57 (1.19–2.07)	0.001
Propensity score	1.23 (1.12–1.67)	0.003	1.01 (1.01–1.02)	0.020

^a^On admission. ^b^On the day of onset of albumin administration in the albumin group and on the day of admission for other patients. CI, confidence interval; SAPS, simplified acute physiology score; SOFA, sequential organ failure assessment.

**Table 5:** Relative risk of albumin administration based upon a Cox proportional hazard analysis^a ^stratified by severe sepsis^b ^and trauma

	n	Relative hazard (95% CI)	*p *value
No trauma	2,893	1.32 (1.09–1.60)	0.005
Trauma	254	2.58 (1.05–6.04)	0.035
No severe sepsis	2,382	1.29 (1.01–1.66)	0.048
Severe sepsis	765	1.01 (1.13–2.00)	0.006

^a^Multivariable, forward, stepwise with time to mortality, right censored at 30 days, as the dependent factor. ^b^On the day of albumin administration in the albumin group and on admission in others.

**Table 6:** Patient characteristics by albumin status for the propensity matched patients

	Albumin (n = 339)	No albumin (n = 339)	*p *value
Age, mean ± SD	62.6 ± 15.1	62.6 ± 17.1	0.365
Male gender (%)	221 (62.2)	206 (60.8)	0.693
Chronic diseases (%)			
COPD	37 (10.9)	33 (9.7)	0.614
Cancer	61 (18.0)	62 (18.3)	0.921
Heart failure	40 (11.8)	43 (12.7)	0.725
Diabetes	27 (8.0)	34 (10.0)	0.347
Liver cirrhosis	30 (8.8)	26 (7.7)	0.577
Hematologic cancer	12 (3.5)	10 (2.9)	0.665
HIV/AIDS	5 (1.5)	7 (2.1)	1.000
Surgical admissions (%)	208 (61.4)	215 (63.4)	0.579
SAPS II score, mean ± SD	41.7 ± 17.2	41.6 ± 18.1	0.664
SOFA score, mean ± SD^a^	7.1 ± 4.1	6.7 ± 4.4	0.126
Organ failure^a^			
Respiratory	106 (31.3)	100 (29.5)	0.616
Hepatic	16 (4.7)	15 (4.4)	0.854
Coagulation	39 (11.5)	27 (8.0)	0.120
Renal	72 (21.2)	71 (20.9)	0.925
CNS	70 (20.6)	79 (23.3)	0.404
Cardiovascular	147 (43.4)	146 (43.1)	0.939
Sepsis syndromes (%)^a^			
Sepsis	120 (35.4)	132 (38.9)	0.441
Severe sepsis	103 (30.4)	101 (29.8)	0.867
Septic shock	57 (16.8)	50 (14.7)	0.461
Procedures (%)^a^			
Mechanical ventilation	259 (76.4)	255 (75.5)	0.720
Pulmonary artery catheter	71 (20.9)	58 (17.1)	0.203
Hemofiltration	23 (6.8)	17 (5.0)	0.335
Hemodialysis	11 (3.2)	9 (2.7)	0.650
Mean fluid balance ± SD	0.2 ± 1.3	0.2 ± 1.4	0.474
Trauma	18 (5.3)	14 (4.1)	0.446
ICU mortality (%)	118 (34.8)	71 (20.9)	<0.001
Hospital mortality (%)	140 (41.3)	94 (27.7)	<0.001

^a^On the day of albumin administration in the albumin group and on admission in the others. CNS, central nervous system; ICU, intensive care unit; IQ, interquartile range; SAPS, simplified acute physiology score; SD, standard deviation; SOFA, sequential organ failure assessment; COPD: chronic obstructive pulmonary disease.
